# Creativity within a military setting: assessing the utility of an existing military visual aid to facilitate military deception amongst a civilian population

**DOI:** 10.3389/fpsyg.2025.1665765

**Published:** 2025-09-26

**Authors:** Callum A. O'Malley, David J. Harris, Tom Arthur, Hannah Blackford, George Raywood-Burke, Nigel Jones, Samuel J. Vine

**Affiliations:** ^1^Department of Public Health and Sport Sciences, University of Exeter, Exeter, United Kingdom; ^2^Trimetis Limited, Bristol, United Kingdom

**Keywords:** cognitive flexibility, creativity, imagination, defense and security, military deception

## Abstract

**Introduction:**

Deception can function as a useful tool for any military figure. Historic examples demonstrate that deception facilitates success by corralling an adversary into failure. Traits such as creativity and imagination are considered of central importance to devising useful and effective deceptive ideas. In lieu of being naturally creative/imaginative, visual aids highlighting core military deception principles could offset these shortcomings. This study assessed whether an existing military deception visual aid improved the number, usefulness, and originality of deceptive ideas amongst a civilian population.

**Methods:**

An independent samples design comprising 80 participants (44 female) were equally assigned to an experimental (with aid) or control (without aid) group. Participants created as many deceptive stratagems in as much detail as possible, during a 15-minute hypothetical task scenario. The number of stratagems and ratings of the usefulness, originality, and a snapshot score of the participant's self-selected best stratagem were compared between experimental groups.

**Results and discussion:**

No significant differences emerged for the number of stratagems (*p* = 0.061, *r* = 0.238) or usefulness (*p* = 0.348, *r* = 0.116), originality (*p* = 0.558, *r* = 0.076), or snapshot scores (*p* = 0.603, *r* = 0.068). Results question whether deceptive thinking for a military context can be improved by a visual aid containing prompts about military deception principles. However, some task elements (e.g., same hypothetical scenario/only rating the best stratagem) may have reduced/nullified potential differences between groups. The use of an existing military deception visual aid may be limited to military samples. Future studies could employ mixed-method approaches or gamified designs to investigate the potential to enhance military deception planning.

## Introduction

Deception has been a central feature of some of the most notorious military events in history. For instance, Operation Fortitude demonstrated the effectiveness of deceptive stratagems whereby false information and battle plans were leaked to the highest levels of enemy command to cause the adversary to falsely anticipate battle strategies that were ultimately wrong. As such, deception is often useful for military situations and therefore, military personnel, regardless of role, could be required to conceive deceptive ideas and put them into action. This could range from elaborate battle strategies to simple concealment of a weapon. However, not all personnel are naturally skilled at deception (Jonason et al., 2014; Wissing and Reinhard, 2019) and, with limited deception training available, visual aids may be useful in assisting the creativity and innovation of ideas. Therefore, this study sought to assess whether an existing military deception visual aid improved the number and quality of deceptive stratagems for a hypothetical task within a civilian population.

Military experts have defined military operations to be a “relationship” between two or more parties in which each party is continually struggling to gain an advantage over the other [Whaley, 1982; UK Ministry of Defence [MOD], 2022]. Most observe military operations in a physical form where relative gain is sought in time and space (Whaley, 1982). However, there is also a prominent cognitive element to military operations wherein multiple psychological components such as motivation, resilience, and cohesion are regularly tested (North Atlantic Treaty Organization (NATO), 2020; UK Ministry of Defence [MOD], 2022; Whaley, 2016). According to the latest North Atlantic Treaty Organization (NATO) (2020) definition, deception within a military context—hereby referred to as “military deception”—involves a deliberate measure to mislead targeted decision-makers into behaving in a manner advantageous to the commander's (deceiver's) intent. In so doing, military deception can create a series of advantages that can be exploited for the attainment of a specific goal (Rothstein and Whaley, 2013). To add, whilst creating advantages, military deception can also promote a more efficient allocation of resources for the physical aspects of military operations (Morrell and Kosal, 2021; Whaley, 2007). Indeed, Sun Tzu's well-known philosophy that “to subdue the enemy without fighting is the acme of skill” attests to this notion.

Subsequently, defense and security organizations are invested in military deception and utilizing it across a range of military contexts (see North Atlantic Treaty Organization (NATO), 2020). As such, personnel, regardless of role, are likely expected to engage with creating deceptive ideas to factor into courses of action during missions (Verrall, 2021). However, the combination of natural inadequacies in military deception (Jonason et al., 2014; Kaufman et al., 2013; Wissing and Reinhard, 2019) and a limited availability of military deception training (Ausseil et al., 2020; Verrall, 2021; Wrangham, 1999) likely means that most personnel are not capable of generating usable and effective deceptive stratagems (Kapoor and Khan, 2017).

Psychological theory also poses two main views about military deception in practice. One view is that military deception is governed by personality traits such as narcissism, psychopathy, and “Machiavellianism,” often referred to as the “dark triad” (Jonason et al., 2014; Wissing and Reinhard, 2019). Subsequently, deception is considered an innate ability that is untrainable. Indeed, Wissing and Reinhard (2019) identified that those who exhibited higher dark triad traits were linked to a better capacity to produce and detect deceptive ideas. Meanwhile, another view is that simple deceptive stratagems can be learned (Rothstein and Whaley, 2013; Skidmore and Ortiz, 2014). Curiously, this viewpoint implies that whilst dark triad traits or other cognitive abilities such as creativity or imagination—be they real or perceived—predispose individuals to creating a greater number or more useful/effective deceptive stratagems (Wissing and Reinhard, 2019), materials that spotlight core military deception principles can easily level the playing field (Ritter and Mostert, 2017; Scott et al., 2004).

In response, materials that compile the scientific principles of military deception into simple, single-sheet visual aids have begun to surface to facilitate deceptive ideas. Within these aids, generic text cues presented in a visually appealing manner via an infographic can calibrate a user's attention to central principles of military deception (Verrall, 2021). For instance, North Atlantic Treaty Organization (NATO) (2020) recently published some core principles of military deception that a user may want to consider: creating a behavioral response; reinforcing existing beliefs; targeting the decision-maker; ensuring ideas are credible, consistent, verifiable, and executable; considering multiple approaches; and concealing the real and revealing the false intentions.

Accordingly, this study sought to test whether an existing military visual aid that outlines core military deception principles improved the number, usefulness, and originality of deceptive ideas that participants with no military experience could generate. It was hypothesized that the group provided with the military deception visual aid (“experimental” group) would generate a higher number of stratagems and that these would also be rated as more useful and original compared to a group without the military deception visual aid (“control” group). We hypothesize that there would be an interaction between participants' self-perceived creativity, cognitive flexibility, and imaginative skills on the main dependent variables (e.g., number, usefulness, and originality of deceptive stratagems) but did not state a direction due to the equivocal findings from previous research in this area.

## Methods

### Participants

A sample of 80 civilian participants (36 male, 44 female) with a mean ± SD age of 21.8 ± 4.2 years was recruited from the student population of the lead author's institution. An a priori calculation at an alpha level of α = 0.05, large effect size: *d* = 0.80, and moderate-high power: β = 0.85 determined that 60 participants would be sufficient to detect meaningful effects on deceptive stratagem ideas (number, usefulness, and originality). The effect size used within the a priori calculation was an average of the two effect sizes from Ritter and Mostert (2017) and Scott et al. (2004). Participants had no pre-existing military experience or psychological conditions that impeded completion of the hypothetical task and spoke/wrote in fluent English. All participants provided written informed consent after receiving information about the study and a cooling period. The study protocol was reviewed by the UK Ministry of Defence Research Ethics Committee (Application number: 2258/MODREC/23). Protocols were completed in accordance with the principles from the Declaration of Helsinki. No participants withdrew from the study at any point, and all received a reimbursement of £20 for their time. The study was registered at https://osf.io/zd8bk/. The study was registered on the 18 March 2024 after data collection had been completed but before any data were analyzed.

### Design

This study employed an independent samples design. Participants were randomly allocated to the experimental or control group upon arrival to the laboratory. Each group/condition consisted of 40 participants each. Participants were not made aware of the full extent of the purpose and hypotheses of the study and were only informed that “the purpose of the study was to better understand how people generate deceptive ideas.” As such, participants were unaware of their group allocation, thus the study employed a single-blind design.

### Protocols

**Figure 1:**
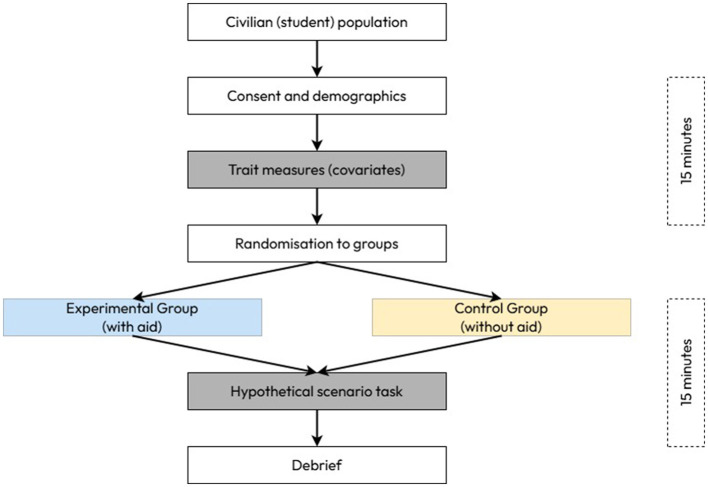
Visual representation of study protocols and timings.

All participants completed a single, 30-min visit to the same laboratory at the lead author's institution (Figure 1). After providing their written informed consent, participants first completed a questionnaire pack (see “questionnaires”) to assess trait measures of cognitive flexibility, creativity, and imagination. After this, the researcher provided a brief explanation of the hypothetical task scenario that participants were to use as a guideline for creating deceptive stratagems (see “scenario” and Supplementary material 1). Participants were then asked to read through the document detailing the hypothetical task scenario without a time limit. For those allocated to the experimental group, the visual aid for military deception was provided. Participants were instructed to “please familiarize yourself with the content of this visual aid in any way you wish and please ask any questions to clarify any content if necessary.” Again, participants were not limited for time to look at the military visual aid and familiarize themselves with the strategic cues provided within.

Finally, once participants were ready, they were asked “to write as many deceptive stratagems as they can in as much detail as they can” during a 15-min period. Prior pilot work with defense and security partners determined that this timeframe imposed a moderate time constraint on the task but also did not restrict creative freedom. Consultations with defense and security staff emphasized that decisions within high-stress, defense, and security settings often demand decision-makers to conceive plans quickly. Last, Silvia et al. (2008) highlight the importance of a time constraint within studies assessing the effects of creativity. Therefore, a 15-min timeframe was considered ideal.

Deceptive stratagems were typed onto a preformatted table within Word (Microsoft: Seattle, WA) that provided some general questions/prompts to guide the details that participants provided (see Supplementary material 2). However, participants were instructed that they “...did not need to fill in every box and could write all their details within one box if they preferred.” Finally, participants were informed that though some of their stratagems might have crossover with one another, they were asked to distinguish between their stratagems however they wished by placing a number in the left-most column to demarcate each stratagem.

If participants thought that they had run out of ideas, they were asked to consider any other ideas and continue to write down anything that came to mind. Participants were made aware of when 5 min and 1 min were remaining to write down their ideas. After writing all their ideas, participants highlighted which stratagem they considered to be their “best stratagem” before a short debrief and exit from the laboratory.

### Scenario

Cultural property protection in the event of armed conflict is a concern of the Hague Convention (1954). Cultural property includes, but is not limited to, art collections, museums, religious sites, and other monuments. In conflict, cultural property can be exposed to deliberate targeting as part of an attack on groups' identity and heritage, looted by criminals and opportunists, or inadvertently damaged. Signatories to the Hague Convention (1954) have a responsibility to take measures to protect it. Past instances have often required the removal of highly valuable and important collections from heritage sites to safe places for the duration of a conflict. In the scenario within this study, participants were informed that they were required to generate stratagems for concealing the timing, method, and arrangements for an evacuation and re-housing of cultural property. Participants were recommended that they should try to conceal the removal before, during, and after transit of the cultural property. This entailed the contribution of both civilian institutional staff and security personnel, with a view to protecting the cultural property from diverse actors, including criminals, spies, warring parties, and/or adversaries. Finally, participants were asked to consider this scenario to be a pre-civil war or invasion context in a fictional state with a fictional institution.

### Deceptive prompts

The experimental groups were provided with phrases via a visual aid that contained simple prompts about deceptive principles: creating a behavioral response; reinforcing existing beliefs; targeting the decision-maker; ensuring ideas are credible, consistent, verifiable, and executable; considering multiple approaches; and concealing the real and revealing the false intentions. This visual aid is derived from previous work (e.g., Verrall, 2021; North Atlantic Treaty Organization (NATO), 2020) which shows that strategic cues of this nature can effectively assist users' deceptive thinking and ultimately help develop deceptive stratagems. Indeed, the visual aid closely reflects the principles outlined within the North Atlantic Treaty Organization (NATO) (2020) doctrine, though more specific details of this aid cannot be shared due to security classification.

### Questionnaires

#### Cognitive flexibility

Cognitive flexibility was assessed via a 12-item Cognitive Flexibility Scale (Martin and Rubin, 1995). Items are rated on a six-point Likert scale ranging from 1 – “strongly disagree” to 6 – “strongly agree.” Total scores ranged from 12, indicating poorer perceptions of cognitive flexibility, to 72, indicating higher perceptions of cognitive flexibility.

#### Creativity

A two-item domain-related creativity survey was adapted from Kaufman et al. (2013). Participants were asked “how creative do you think you are when it comes to planning?” which was rated on an inverted five-point Likert scale ranging from 1 – “very” to 5 – “not at all.” Participants were also asked “to what extent do you feel that you have good imagination when creating plans” which was rated on an inverted five-point scale from 1 – “very much so” to 5 – “not at all.” Total scores ranged from 2, indicating higher perceptions of creativity, to 10, indicating lower perceptions of creativity.

#### Imagination

Imagination was assessed via a 25-item Self-Descriptive Imagination Questionnaire (Feng et al., 2017). Items are rated on a seven-point Likert scale ranging from 1 – “not at all” to 7 – “absolutely.” Total scores ranged from 25, indicating lower perceptions of imagination, to 175, indicating higher perceptions of imagination.

### Stratagem ratings

In accordance with creativity literature (e.g., Kapoor and Khan, 2017), participants best stratagems were assessed according to their volume (number/frequency of stratagems); usefulness; and originality. In addition, a snapshot metric score (Equation 1) was calculated as a general effectiveness score that combined the usefulness, originality, and fluency of the deceptive stratagem selected as the best by the participant.


(1) $$\begin{array}{l} number\;of\;stratagems\times usefulness\times originality \end{array}$$

The pre-registered rating rubric (see Supplementary material 3) assessed stratagem usefulness on a five-point scale ranging from 1 – “not at all useful” to 5 – “very useful.” Stratagem originality was comprised of three subcomponents: frequency in sample, remoteness, and cleverness. Originality is considered as being akin to creativity, which itself is a function of being uncommon, remote, and clever (Silvia et al., 2008). Frequency within sample was assessed within each batch (collection of 10 participant stratagems). The rubric was originally designed by an expert with military experience who used content from prior deception-based publications (e.g., Kapoor and Khan, 2017; Verrall, 2021; Whaley, 2007; North Atlantic Treaty Organization (NATO), 2020) that discuss the importance of measurable aspects of deception (e.g., fluency, usefulness, originality, and effectiveness). The initial draft of this rubric was provided by an expert in a workshop that included familiarization to the rubric and a training exercise for all other raters. Part of the training exercise in this workshop involved the raters completing a mock rating of a subsample of participants' stratagems. At the end of this exercise, the lead researcher completed an inter-rater reliability assessment that showed that raters understood the content of the workshop and rated stratagems reliably. Specifically, raters 1 and 4 (lead researcher and expert), who would be the primary raters of the stratagems, did not show a significant difference in ratings to each other. In a second meeting, aspects of the rubric were discussed by all of the team, which included cognitive psychologists and military practitioners. During this discussion, minor iterative changes were made to some of the phrasing of the rubric. The final version of the rubric was confirmed by all authors. Collectively, the authors were satisfied with the rubric based on the agreement between all raters, preliminary inter-rater reliability checks, and consistency with published literature. Supplementary material 4 depicts which stratagems were assessed by which rater and the comparisons made as part of the interrater reliability assessments.

### Analysis

All data including number of stratagems and researcher ratings were input into JAMOVI software (JAMOVI, Sydney, Australia) and assessed for normality using a Shapiro-Wilk test and visual inspection of Q-Q plots. Interrater reliability of stratagem ratings was assessed via two-way, random effects, intra-class correlation coefficient model. Intraclass correlation coefficients were interpreted as ≤ 0.500—poor reliability; 0.500 to 0.699—moderate reliability; 0.700 to 0.899—good reliability; and ≥0.900—excellent reliability according to previous research (Koo and Li, 2016).

Assuming parametrically distributed data, the number of stratagems created and the best stratagem rating from Rater 1 (military deception expert) and Rater 4 (primary investigator) were used in subsequent independent samples *t*-tests. Non-parametric data were analyzed via a Mann Whitney U-test for between group comparisons. Data were assessed according to an alpha level of 0.05 and Cohen's *d* (parametric) or a rank biserial correlation (non-parametric); effect sizes were calculated with 95% confidence intervals. Additional analysis of covariance (ANCOVA) tests was performed by adding the participant's self-reported cognitive flexibility, creativity, and imagination scores. Though researchers expected small deviations from normality due to ratings on a Likert scale (Norman, 2010), ANCOVAs are typically robust enough to factor in some deviations. Effect sizes for ANCOVAs were calculated as partial eta squared ($\eta_{p}^{2}$) values and interpreted as ≥0.01 to 0.09 – small, 0.10 to 0.24 – moderate, and ≥0.25 – large.

## Results

### Standardization

Perceived cognitive flexibility [*t*_78_ = 0.325, *p* = 0.746, *d* = 0.073, 95*%CI* (−0.366, 0.511) ], creativity (*U* = 752, *p* = 0.638, *r* = 0.061), and imagination [*t*_78_ = −1.241, *p* = 0.218, *d* = 0.278, 95*%CI* (−0.718, 0.167)] were not significantly different between the participants of each condition and all with trivial to small effect sizes. Table 1 provides mean ± SD of all participants' perceived cognitive flexibility, creativity, and imagination scores.

**Table 1:** Mean ± SD total scores for participants' cognitive flexibility, creativity, and imagination questionnaires.

**Condition**	**Cognitive flexibility**	**Creativity**	**Imagination**
Experimental	48.3 ± 2.78	5.45 ± 1.52	110 ± 17.1
Control	48.1 ± 3.38	5.58 ± 1.74	115 ± 14.9

### Interrater reliability

Table 2 matrix provides the intraclass correlation coefficient outputs for stratagem usefulness, originality, and snapshot scores. There was moderate to excellent reliability between researchers for originality and snapshot score ratings. However, there was a poor to moderate reliability between researcher ratings for usefulness.

**Table 2:** Interrater reliability intraclass correlation coefficient outputs for subjective ratings of usefulness, originality, and snapshot score of participants' best stratagems.

	**Rater comparison**			
**Variable**	**1–2**	**1–3**	**4–5**	**4–6**
Usefulness	0.537	0.274	0.213	0.595
Originality	0.937	0.607	0.918	0.582
Snapshot score	0.628	0.860	0.637	0.847

### Condition effects

**Figure 2:**
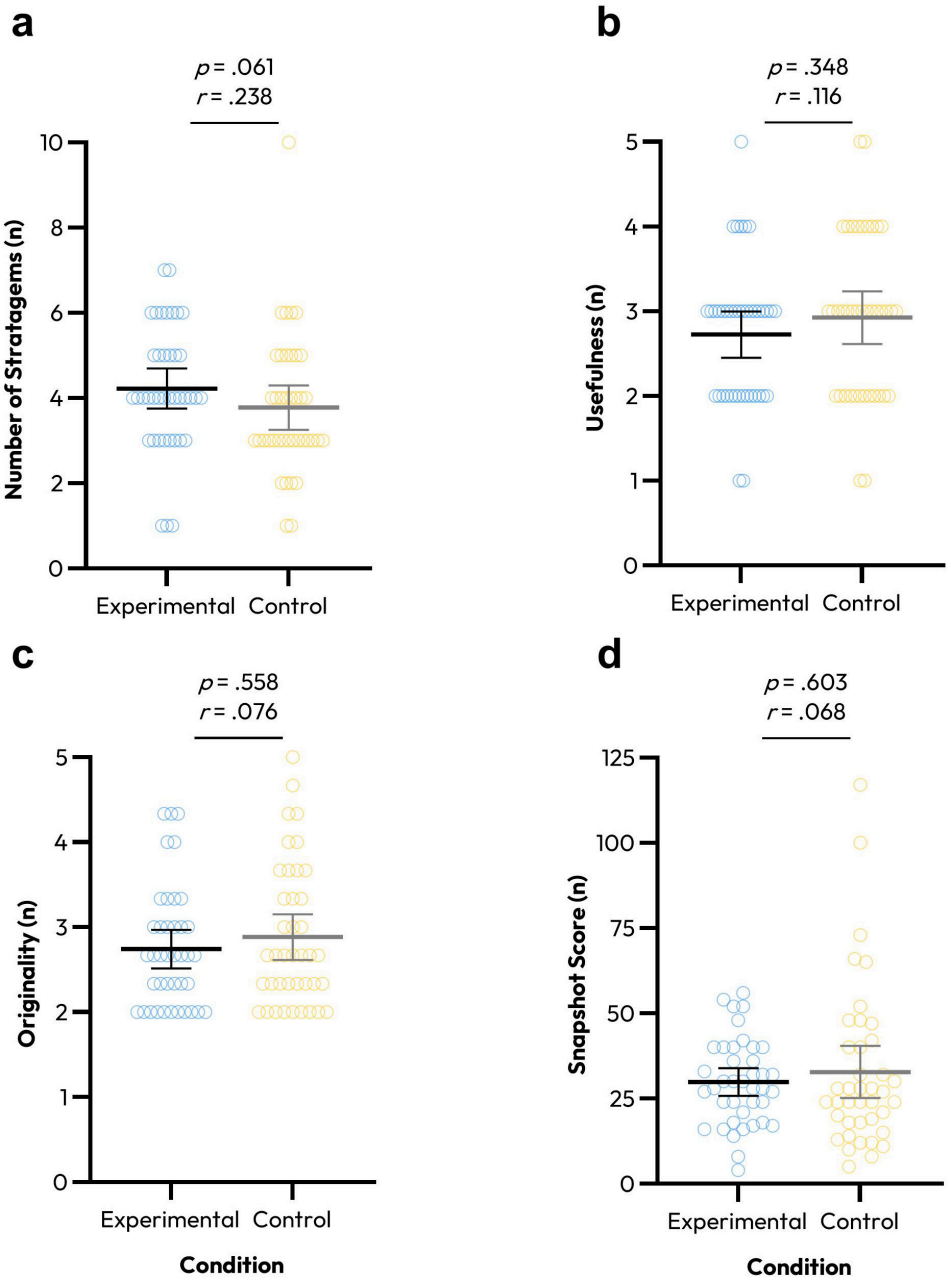
Individual plots (hollow circles) of researcher ratings from the experimental hypothetical task scenario of the participant's total number of stratagems **(a)** and the usefulness **(b)**, originality **(c)**, and snapshot score **(d)** of participant's self-selected best stratagems from the experimental (blue) and control (yellow) conditions. Lines and error bars represent mean ± 95% confidence intervals.

There was no significant difference between the number of stratagems generated between conditions (*U* = 610, *p* = 0.061, *r* = 0.238); there was a slightly higher number of stratagems generated on average within the experimental (4.2 ± 1.48) vs. control (3.77 ± 1.64) condition but with small, insignificant effects (Figure 2a).

There was no significant difference between conditions for the subjective usefulness (*U* = 708, *p* = 0.348, *r* = 0.116), originality (*U* = 740, *p* = 0.558, *r* = 0.076), or snapshot score (*U* = 746, *p* = 0.603, *r* = 0.068) of participant's best stratagems as rated by researchers (Figures 2b–d). In addition, there was no significant difference between conditions for the ratings of the subcomponents of originality such as frequency in sample (*U* = 709, *p* = 0.353, *r* = 0.114), cleverness (*U* = 764, *p* = 0.701, *r* = 0.0046), or remoteness (*U* = 764, *p* = 0.712, *r* = 0.045).

One ANCOVA assessment observed a small condition × imagination effect on subjective originality ratings of participant's best stratagems as rated by researchers $\left(F=4.54,\;p=0.036,\;\eta_{P}^{2}=0.056\right)$. From this, it appears that participants allocated to the control condition had slightly higher originality scores in the control than experimental condition, possibly affected by the perceived imagination scores of participants. All other ANCOVA assessments did not detect any effect of perceived cognitive flexibility, creativity, or imagination scores on any subjective usefulness, originality, or snapshot scores of participant's best stratagems as rated by researchers. Example participant stratagems have been provided in Supplementary material 5.

## Discussion

The present study sought to test whether prompts about deceptive principles via a military visual aid improved the number, usefulness, and originality of deceptive ideas that participants with no military experience could generate. Results provide a reliable empirical case that providing participants with an outline of core military deceptive principles via an existing visual aid does not facilitate more numerous or effective deceptive ideas as there were no significant differences between the number of stratagems created or subjective ratings of usefulness, originality, or a snapshot score of participant's best stratagems. Furthermore, ANCOVA assessments largely did not observe any significant effects of perceived cognitive flexibility, creativity, or imagination on these subjective ratings. Therefore, it appears that the provision of a visual aid that includes details of military deception principles did not facilitate deceptive thinking when accounting for varying perceptions of cognitive flexibility/creativity/imagination amongst civilian participants. However, one small condition × imagination effect was significant when assessing originality ratings. That is, as perceived imagination scores were slightly, but not significantly, higher amongst the participants of the control vs. experimental condition (Table 1), when controlled for condition, imagination scores may be indicative of slightly higher originality ratings of participants' best stratagems. Altogether, the results of this study question whether deceptive thinking in a military context can be improved by a series of prompts outlining military deceptive principles housed within an existing visual aid.

One area of caution is that a small portion of the interrater reliability values were low for certain measures and between certain raters of the present study (Table 2). To reiterate, all raters completed their ratings in a single-blinded fashion according to a standardized rating rubric (Supplementary material 3). The rating rubric was created by the research team, which included a mixture of cognitive psychologists and individuals with experience in military deception during a practice assessment of a subset of the participant data. Notably, the rating rubric was piloted by all the raters and analysis for consistency before any final analysis of the study. Thus, it is unexpected that assessments of interrater reliability were low in some cases. Relatedly, as the ratings of stratagems are, to some degree, subjective, it may be that meaningful differences between conditions could have been undetected. However, several elements of the research process make this unlikely. One, this study was of high statistical power whereby a large sample was recruited. Second, the researchers took numerous precautions and steps to pilot and trial the ratings aspect of the study. Third, though some raters may have showed lower reliability compared to others, crucially, the ratings from the principal investigator and a member of the research team with experience of using military deception stratagems *in-situ* were used for statistical analysis between conditions, meaning that ratings from “less reliable” raters were discounted at this point.

Beyond the specific military visual aid, it may be that other aspects of the research protocol prompted the null findings of this study. To illustrate, the hypothetical scenario provided all participants with a standardized task to which they were to generate deceptive stratagems. As such, it may have naturally disposed participants within both conditions to have generated similar ideas (Wirtz, 2023) that were equally effective at attaining the task goal but were discounted against one another in some aspects of the ratings that were used (Kapoor and Khan, 2017). Consequently, the potential differences that strategic cues could evoke may have been dampened or nullified. Nevertheless, deception in the military does not occur in a vacuum (Whaley, 2007), and adversaries are equally capable of detecting deceptive stratagems against themselves and utilizing counter-deception in response (Skidmore and Ortiz, 2014; Wirtz, 2023). Therefore, whilst this task scenario may have disposed participants across conditions to formulate similar ideas, this is reflective of the real world wherein the most original ideas out of hundreds of suggestions often translate into the most useful and effective (Morrell and Kosal, 2021; Rothstein and Whaley, 2013).

However, one should be wary of completely discounting the efficacy of outlining core military deceptive principles to assist deceptive idea generation and, therefore, the notion that military deception is trainable. In some respect, the purpose of military deception is not always to dupe an adversary and meet a fixed goal *immediately* (Wirtz, 2023). Alternatively, deception has also been posited as a means to *gradually* demoralize or diminish a counterparts' will or fighting power (Rothstein and Whaley, 2013; Whaley, 1982, 2007; Wirtz, 2023) and that often deception requires time to “mature” (North Atlantic Treaty Organization (NATO), 2020). Therefore, the immediate usefulness or originality of deceptive stratagems may not be explicit (Kapoor and Khan, 2017). By contrast, this study was solely focused on assessing the immediate use, originality, and overall effectiveness of a deceptive stratagem that participants had selected as their best idea through quantification of qualitative data. As a result, some of the more implicit nuances within the qualitative data about military deception may have been underestimated and not accounted for within this study's assessment.

In addition, there is likely a slight disconnect between empirical scientific accounts of which deceptive stratagems are useful or original and the true effectiveness of a stratagem within a real-life military scenario (Ausseil et al., 2020). Several infamous accounts of deception in real-life settings (e.g., Operation Mincemeat, Argo exfiltration) on a theoretical level would be considered almost too outrageous to be useful or effective. However, the novelty of these operations is perhaps what made them so effective in practice. Therefore, whilst this study is one of the first to aim to employ a robust, empirical assessment of deception ideas that is predicated on previous scientific findings and approaches (e.g., Kapoor and Khan, 2017; Kaufman et al., 2013; Ritter and Mostert, 2017; Scott et al., 2004; Silvia et al., 2008, 2009), it may be that more reflexive approaches (see Braun and Clarke, 2019) that incorporate a mixture of qualitative and quantitative measures and discard prior assumptions could discern more about how to train deceptive thinking and promote idea generation.

Accordingly, future research may wish to consider some of these methods. In particular, it would be interesting to see more research involving a controlled manipulation of exposure to select deceptive principles via visual aids or other contextual changes in tandem with mixed method approaches that delve more into qualitative accounts of how participants generate deceptive ideas and the logic/purpose behind them. Moreover, it would also be worthwhile exploring the effect of deceptive aids within group settings to uncover how several members of a team can conjure more ideas based on prompts from their colleagues. Finally, if future research is intent on using a similar empirical and scientific approach to this study, alternative means of assessing the quality and effectiveness of deceptive ideas may be beneficial. For example, compared to a series of Likert scales, a discounting scoring system could provide an interesting account of which stratagems/ideas are original (c.f. our definition of “original” in the Methods section above). To add, the gamification of deceptive scenarios could also provide a unique insight into the use and effectiveness of ideas to assess whether they truly cause opponents/adversaries (e.g., within the game) to act in a desired manner.

## Conclusion

The present study did not exhibit any significant benefits to generating more useful, original, or effective deceptive ideas when provided with existing military visual aid that outlined core military deception principles vs. a control (no aid). As such, this study casts doubt as to whether outlining core deceptive principles via an existing visual aid is advantageous for creating more useful, original, and/or effective deceptive ideas for a military-related scenario. Additional covariate analysis showed relatively little interacting effects of existing personal factors such as cognitive flexibility, creativity, and imagination on the production of more useful, original, or effective deceptive stratagems. However, this study reasons that null results may have been a product of the quantification of qualitative data or the civilian sample that was used. It is conceivable that alternative study designs that recruit participants with a military background and employ reflexive mixed-method approaches may generate different results about the utility of military deception aids. Furthermore, “darker” personal factors like narcissism, psychopathy, and Machiavellianism were not considered in the present study, though some evidence suggests they may underpin an individual's capacity for effective deception within similar situations. Therefore, we invite future research to explore various areas relating to the “light” and “dark” aspects of individual's creativity, particularly in relation to the nature, feasibility, and ethics of deception with defense and security settings.

## Data Availability

The datasets presented in this study can be found in online repositories. The names of the repository/repositories and accession number(s) can be found below: https://osf.io/zd8bk/.
