# Supplementary material for: Creativity within a military setting: assessing the utility of an existing military visual aid to facilitate military deception amongst a civilian population
Source: Front Psychol. 2025 Sep 26;16:1665765. doi: 10.3389/fpsyg.2025.1665765 (PMC12510928; doi:10.3389/fpsyg.2025.1665765)
Supplement: Supplementary file 1 [file Data_Sheet_1.pdf]

Scenario

There has been a serious escalation in the threat to an allied country by another country. If conflict breaks out as feared, it may well involve attacks against art collections, museums, religious sites and other monuments. These locations are seen by the hostile adversary as symbols of the allied country's national identity.

You have been asked to support a team by generating ideas for how you might deceive a potential attacker or criminal regarding the preparation, movement, and relocation of the cultural property to a predesignated safe storage facility. Of the several thousand items in the gallery, there are over 100 paintings and sculptures (many quite large) that are seen to be of the highest importance globally. The overall plan is to move the contents of the gallery to a secret underground storage location for safe keeping. This facility is already being prepared some 300 miles away from the gallery. There are two essential desired outcomes for this activity:

1. The adversary does not discover the location of the storage facility.
2. The chances of the art being attacked (or indeed stolen) are reduced as much as possible during preparation and transit.

You have been asked to think about one element of this activity, which is to generate ideas for how deceptive actions might support these two essential outcomes.

Please treat this scenario as if you always have the required resources available to enact your stratagems.

For information, Cultural Property Protection in the event of armed conflict is the concern of the Hague Convention 1954, to which the UK is a signatory. Cultural property has been targeted deliberately or accidently damaged throughout the history of conflict. Evacuation of cultural property has consequently also been a feature of preparing for conflict, as was the case in the museums and galleries of London, Paris and Amsterdam in the Second World War. There is in fact UNESCO guidance on good record keeping, packing and safe removal of museum contents when being evacuated in times of conflict, all of which help to ensure the identification, tracking, maintenance and environmental protection of valuable items.
