# Supplementary material for: Creativity within a military setting: assessing the utility of an existing military visual aid to facilitate military deception amongst a civilian population
Source: Front Psychol. 2025 Sep 26;16:1665765. doi: 10.3389/fpsyg.2025.1665765 (PMC12510928; doi:10.3389/fpsyg.2025.1665765)
Supplement: Supplementary file 2 [file Data_Sheet_2.pdf]

Participant ID: ____________________

During this task you will be presented with a scenario and asked to create deceptive stratagems to fulfil the aims of the scenario. You will have **15 minutes** of time to create deceptive stratagems**.**

Please provide as many stratagems as you like with as much detail as you wish.

Each time you feel you have provided all the detail you want for a given stratagem, please move onto the next row down/draw a line to then begin detailing your next deceptive stratagem. If you run out of room, extra space is provided for you to continue disclosing your stratagems.

At the end of the task, you will be asked to circle or highlight what you consider to be your **best** stratagem.

**Stratagem**	What are you intending to do?	What actions would you take?	What are the details of what you are doing? (e.g., How and Why?)
1			

Participant ID: ____________________

PLEASE IDENTIFY YOUR **BEST** STRATAGEM FOR ACHIEVING THE SCENARIO GOAL

PLEASE CIRCLE/HIGHLIGHT **ONLY ONE** STRATAGEM YOU HAVE LISTED
