# Supplementary material for: Creativity within a military setting: assessing the utility of an existing military visual aid to facilitate military deception amongst a civilian population
Source: Front Psychol. 2025 Sep 26;16:1665765. doi: 10.3389/fpsyg.2025.1665765 (PMC12510928; doi:10.3389/fpsyg.2025.1665765)
Supplement: Supplementary file 3 [file Data_Sheet_3.pdf]

Deceptive Stratagems Rating Rubric

Usefulness *(how useful is the stratagem in achieving its goal?)*

Score	1<br>**(Not at all useful)**	**2**	**3**	**4**	**5**<br>**(Very useful)**
Detail	Not linked to mission success<br><br>No perceivable logic or theory of change<br><br>Completely unviable	Remote link to mission<br><br>Unpragmatic	Linked to mission success<br><br>and<br><br>Has a perceptible logic or theory of change, even if flawed<br><br>Limited viability	Linked to mission success<br><br>Demonstrates a logic or theory of change<br><br>Could be viable	Has two/3 elements to be very useful:<br><br>1. Clearly aligned with achieving the mission<br><br>2. Has an explainable logic, demonstrating a clear theory of change<br><br>3. Clearly viable? Pragmatic?

Originality *(How novel is the stratagem?)*

Score		1 (Very unoriginal)	2	3	4	5 (Very original)
Detail[1]	Frequency in sample[2]	Very common	Common	Mean/ mode/ median?	Uncommon	Very Uncommon
	Remoteness/	Manipulating obvious factors without any clear deceptive intent.	Manipulates obvious factors with deceptive intent, even if not contributing to the mission	Contributes to the mission manipulating obvious factors	Contributes to the mission whilst manipulating tangential factors to the matters at hand	Contributes to the mission by manipulating factors with no obvious connection to the matters at hand
	Cleverness	Demonstrates an absence of characteristics that are considered cunning, insightful, smart, fitting, humorous or ironic in tone	Shows limited cunning and insight, smartness, humour or irony etc.	A fitting idea	A fitting idea that shows insight and some cunning.	Clearly demonstrates cunning or guile[3] with characteristics that are insightful, smart, fitting, humorous or ironic etc.

[1] Based in 'Originality being akin to Creativity', itself a function of an being uncommon, remote and clever. Silvia et al, (2007) Assessing Creativity With Divergent Thinking Tasks: Exploring the Reliability and Validity of New Subjective Scoring Methods.
[2] Assessment of the frequency within the 'batch' (collection of 10 stratagems) that are being assessed.
[3] I've added Guile and Cunning to these characteristics as properties associated with clever deception...

1. Uncommon Creative ideas are uncommon: they will occur infrequently in our sample. Any response that is given by a lot of people is common, by definition. Unique responses will tend to be creative responses, although a response given only once needn't be judged as creative. For example, a random or inappropriate response would be uncommon but not creative.

2. Remote Creative ideas are remotely linked to everyday objects and ideas. For example, creative uses for a brick are —far from common, everyday, normal uses for a brick, and creative instances of things that are round are —far from common round objects. Responses that stray from obvious ideas will tend to be creative, whereas responses close to obvious ideas will tend to be uncreative.

3. Clever Creative ideas are often clever: they strike people as insightful, ironic, humorous, fitting, or smart. Responses that are clever will tend to be creative responses. Keep in mind that cleverness can compensate for the other facets. For example, a common use cleverly expressed could receive a high score.
