# Supplementary material for: Creativity within a military setting: assessing the utility of an existing military visual aid to facilitate military deception amongst a civilian population
Source: Front Psychol. 2025 Sep 26;16:1665765. doi: 10.3389/fpsyg.2025.1665765 (PMC12510928; doi:10.3389/fpsyg.2025.1665765)
Supplement: Supplementary file 4 [file Data_Sheet_4.pdf]

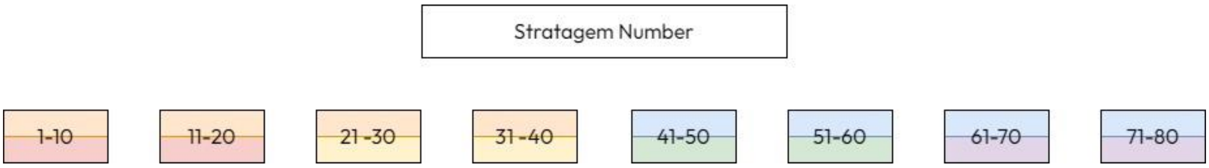

Supplementary Materials 4. Visual representation of rating strategy. Stratagem numbers are shaded within a colour that represents a specific rater: Orange – rater 1; Red – rater 2; Yellow – rater 3; Blue – rater 4; Green – rater 5; Purple – rater 6. Two tone of each number range represents the stratagems which were compared in the interrater reliability assessments for each rater.
