# Supplementary material for: Creativity within a military setting: assessing the utility of an existing military visual aid to facilitate military deception amongst a civilian population
Source: Front Psychol. 2025 Sep 26;16:1665765. doi: 10.3389/fpsyg.2025.1665765 (PMC12510928; doi:10.3389/fpsyg.2025.1665765)
Supplement: Supplementary file 5 [file Data_Sheet_5.pdf]

Example deceptive stratagems of varying originality and usefulness.

	Originality	Usefulness
Low	"Send property to other consulates in other countries… Use other trusted and or friendly countries to take the property and store them safely".	"The person moving the property is going to find three people to assist and a lorry. They are then going to pack up the belongings and move them into the truck. This is going to happen around the back door, away from the public. The idea here is that they are seen moving furniture rather than stealing it."
Moderate	"Begin preparation for movement of dummy cultural items in a way that appears genuine to espionage… …Send [the] task to [a] government department to oversee this. Get a junior minister appointed to oversee the movement of the dummy artefacts… …[This] should [make the act] appear genuine as this is a civil matter. [By having a] government department not ideally set up for counter espionage in charge would conceal that they are moving dummy items".	"Create some 'fake' items and send out information on the location/transportation of these that would be intercepted by the 'enemy'. mask the 'real' items by hiding them as different / fewer valuable items and transport separately".
High	"…Set up a number of Just Stop Oil protests across the country, dotted in random places, including in cities and in the countryside. The protests will take place on statues across the country, the focus on the media will therefore be on these statues and will take away from the paintings and sculptures in the gallery… causing such uproar on social media sites there will be very little focus on the movement of these art pieces from the gallery. Also, because there will be so much media coverage it will seem obvious that the art would not be moved today however the media will be controlled, and only showing parts of the protests in the areas that are not part of the movement of art from the gallery"	"As many transportation methods as possible will be used to minimise risk of prolonged travel that would be suspicious and traceable. This plan is based off the concept of hiding in plain sight. Main transportation will be that of public means (i.e., commercial airlines) as opposed to private to allow for this. Objects will be moved within the same time period with decoys departing simultaneously as a precaution. Realistic decoys of each object should be made, with even those transporting unaware of the object they are transporting let along if it is a decoy or not. All objects, decoy or otherwise will arrive at the safe location and stored appropriately, using the decoys advantageously should the safehouse be invaded. All personnel involved will have the highest clearance and all lines of communications will be clean to maintain the highest levels of secrecy. I would implement spies into the enemies forces to inform me of their knowledge on these objects and their transportation. This will enable a plan to be derived under safety of secrecy. By releasing information in plain sight concealed within speculation, the enemy would have to use extensive resources and time to identify the true nature of transportation and safekeeping"
